# Breastfeeding practices and associated factors at the individual, family, health facility and environmental levels in China

**DOI:** 10.1111/mcn.13002

**Published:** 2020-06-30

**Authors:** Jia Li, Tuan T. Nguyen, Xiaobei Wang, Roger Mathisen, Jin Fang

**Affiliations:** ^1^ Institute of Economics, School of Social Sciences Tsinghua University Beijing China; ^2^ Alive & Thrive Southeast Asia FHI 360 Hanoi Vietnam; ^3^ China Development Research Foundation Beijing China

**Keywords:** breastfeeding duration, breastfeeding initiation, breastfeeding knowledge, breastfeeding support, China, infant formula

## Abstract

We examined the association between breastfeeding practices and associated factors using cross‐sectional data from face‐to‐face interviews with 9,745 mother–child dyads in China. The study collected information on breastfeeding practices and potential associated factors at the individual, family, health facility and environmental levels in China. We used survey commands in Stata to consider sampling weight and survey design effects. Although breastfeeding was the norm (97.4% ever breastfed), the prevalence of early initiation of breastfeeding (EIBF) in 0–11 months old was 8.2%, exclusive breastfeeding (EBF) in 0–5 months old was 27.8% and breastfeeding on the previous day in 6–11 months old was 77.5%. The prevalence of EIBF was lower for caesarean delivery and among mothers belonging to ethnic minority groups. The prevalence of EBF was higher among mothers who practiced EIBF, received information that encouraged breastfeeding and knew that a baby should be breastfed on demand and exclusively. By contrast, the prevalence of EBF was lower in mothers who received infant formula advice or felt uneasy breastfeeding in public places. The prevalence of breastfeeding on the previous day was higher among mothers whose partners supported breastfeeding and who knew about timing of colostrum production, EBF for 6 months, and to nurse more to stimulate milk production. The prevalence of breastfeeding on the previous day was lower in mothers who received infant formula advice or felt uneasy breastfeeding in public places. In conclusion, we found that the prevalence of EIBF and EBF practices in China was low and associated with factors at individual, family, health facility and environmental levels.

**Key Messages**

- Few large‐scale studies have examined factors associated with poor breastfeeding practices in China comprehensively.
- This study, covering seven regions in China, shows a low prevalence of recommended breastfeeding practices.
- Reported barriers to exclusive breastfeeding (EBF) were feeding the infant formula and giving water, limited breastfeeding knowledge, infant formula promotion, early return to work, limited workplace support and lack of public breastfeeding rooms.
- Timely investments in a comprehensive EBF programme are warranted for China to meet the World Health Assembly Target to increase the rate of EBF up to at least 50% by 2025.

## 1 INTRODUCTION

Despite their established benefits, breastfeeding practices remain suboptimal worldwide: the global prevalence of early initiation of breastfeeding (EIBF), exclusive breastfeeding (EBF) and continued breastfeeding at 2 years is less than 50% (Global Nutrition Report, 2018; Rollins et al., 2016; UNICEF, 2019). The annual increase in the prevalence of EBF was just 5 percentage points in the last 14 years (from 35% in 2005 to 42% in 2018). In contrast to the global trend, the prevalence of EBF in China declined from 27.6% in 2008 (Center for Health Statistics and Information at Ministry of Health of People's Republic of China, 2009) to 20.7% in 2013 (Duan et al., 2018). The annual economic cost of not breastfeeding according to recommendation has been estimated to be US$ 66.1 billion or 0.61% of China's gross national income (Walters, Phan, & Mathisen, 2019).

Various factors are associated with breastfeeding practices in China, such as the level of education, employment status, ethnicity and family income (Duan et al., 2018; Gao et al., 2016; Li, Li, Ali, & Ushijima, 2003; Xu et al., 2007) as well as mothers' nutrition knowledge (Gao et al., 2016; Guldan et al., 1993; Kong & Lee, 2004; Shi, Zhang, Wang, & Guyer, 2008). These studies focused primarily on socio‐economic and individual‐level factors in specific geographic areas with the exception of a study by Duan et al. (2018). As suggested by Rollins et al. (2016), breastfeeding practices are also associated with factors related to health care systems, families, workplaces, the built environment and the marketing of breastmilk substitutes (BMSs).

In the last 50 years, China has achieved significant economic growth and development and became the second largest economy in the world (The World Factbook 2019, 2019). Yet this growth has not been accompanied with strengthening policies to support breastfeeding. For example, although about 70% of women in China participate in the labour force—among the highest in the world (Ye & Zhao, 2018)—maternity protection entitlements are limited. Paid maternity leave covers 14 weeks (General Office of the State Council of the People's Republic of China, 2012), meeting only the minimum standard in the Maternity Protection Convention No. 183, much lower than 18 weeks recommended by the Maternity Protection Recommendation No. 191 (International Labour Organization, 2014). It also does not align with the recommended EBF duration of 6 months. In addition, China is the largest market for BMSs globally (Rollins et al., 2016) and since the end of 2017, has no effective national legislation that aligns with the International Code of Marketing of BMS (the Code; National Health and Family Planning Commission of the People's Republic of China, 2017).

Adequate nutrition, health and the well‐being of populations are essential for sustained growth and development (UNICEF, 2019). To contribute to informing efforts to effectively promote, protect and support breastfeeding in China, a better understanding of the factors influencing breastfeeding practices is needed (UNICEF, 2019). However, few large‐scale studies have been conducted in China that examine the complex determinants of breastfeeding practices. To address this gap, we conducted a large‐scale study to examine the association between breastfeeding practices and factors at the individual, family, health facility and environmental levels in China.

## 2 METHODS

### 2.1 Participants and data collection

This study is based on data from 10,408 mother–child dyads who participated in a survey on factors influencing breastfeeding in China conducted in 2017–2018. This study was a part of the Breastfeeding Promotion Initiative at the China Development Research Foundation (CDRF). The main mission of the CDRF is to improve the policy environment and address cultural norms affecting breastfeeding in China. The study was designed, administered and implemented by an independent research institute—the National Institute for Nutrition and Health at the Chinese Center for Disease Control and Prevention (NINH, China CDC). The sample size calculation was based on the prevalence of EBF rate for infants under 6 months of 20.8% (Yang et al., 2016), precision of 4%, design effect of 2 and an estimated 10% of nonresponse rate for 12 strata.

A multistage, stratified cluster sampling approach was used for the selection of the survey sample. We selected 12 districts/counties from all seven regions and included 12 of 34 provincial‐level administrative divisions considering population size, executive capacity and collaboration of the provincial‐level CDC in China. These districts/counties represented three strata: large cities (four districts/counties), medium and small cities (four districts/counties) and rural areas (two districts/counties in general rural areas and two in rural poor areas). One county in rural areas was purposely selected to provide baseline data for a CDRF project intervention.

In each selected district/county, the researchers at NINH, China CDC obtained the list of all clusters and their population size using data provided by the provincial‐level CDC. Four clusters were randomly selected from the list via PPS. In cities, a cluster was typically equal to a subdistrict. Small‐size subdistricts in rural areas were combined to form a cluster. In each selected cluster, the data collection team visited the corresponding immunization clinic and invited mothers who brought their 0‐ to 11‐month‐old children to the clinic for immunization and to participate in the study. With written consent of a mother, the interviewer proceeded with the questionnaire. The process continued until the sample size reached 210 mother–child dyads; about 50% were mothers of children 0–5 months.

Training for technical experts, supervisors and interviewers followed by data collection took place between September 2017 and January 2018 by 12 research teams. Each team included CDC staff at national, provincial and district/county levels. Staff at the national and provincial levels served as trainers and supervisors, whereas CDC staff at the district/county level were interviewers. Local collaborators helped to connect interviewers with survey participants. Before the data collection, the research experts from the NINH, China CDC trained the field supervisors and technical experts in the 12 districts/counties who in turn trained the district/county teams. Selected interviewers had extensive experience and skills in conducting similar surveys because most of them were members of the surveillance team of the local CDC. Training materials and implementation and quality control procedures were standardized to ensure consistency in data collection. Researchers at the NINH, China CDC developed and tested the structured questionnaire and programmed it for recording data in a smartphone or tablet. The survey team collected information through face‐to‐face interviews and recorded it on their mobile devices. The data were immediately uploaded to the server. Researchers at the NINH, China CDC routinely monitored the data to ensure data quality and survey progress.

### 2.2 Study variables

#### 2.2.1 Outcome variables

Infant and young child feeding practices were assessed using indicators recommended by the World Health Organization (WHO), based mainly on foods and drinks consumed the previous day and night (Daelmans, Dewey, & Arimond, 2009). The following indicators and descriptions were used: (i) *Children ever breastfed—*Proportion of children born in the last 12 months who were ever breastfed; (ii) *EIBF*—Proportion of children born in the last 12 months who were put to the breast within 1 h of birth; and (iii) *EBF under 6 months*—Proportion of infants 0–5 months old who were fed exclusively with breastmilk in the previous 24 h (no foods, no liquids except for medications such as drops and syrups). In this study, we used *receiving any breastmilk on the previous day* as a proxy indicator for the continuation of breastfeeding among infants 6–11 months old.

#### 2.2.2 Exposure variables

Correct latching was defined when mothers recalled that they kept their babies latched before their milk came in. Questions related to breastfeeding included five topics: (1) number of days after birth, a mother still produces colostrum (*within 7 days*); (2) the duration a child should be exclusively breastfed (*6 months*); (3) the best way to stimulate milk production (*nurse more*); (4) how often to feed a baby (*on demand*); and (5) awareness of the benefits of breastfeeding. The benefits of breastfeeding included 10 items: (5.1) helps uterus and other organs to return to normal; (5.2) helps mothers to regain a desired figure and reduce weight; 5.3) lowers the risk of ovarian and breast cancer; (5.4) helps to delay menstruation; (5.5) more economical and safer than infant formula; (5.6) can meet the baby's physiological needs; (5.7) promotes baby's emotional and intellectual development and strengthens the connection between baby and mother; (5.8) helps to reduce the risk of being overweight, obese, and developing chronic diseases; (5.9) helps to build up immunity and reduce the risk of infectious diseases and (5.10) reduces the likelihood of babies having an allergy. For the analysis, we generated a score for the awareness of the benefits of breastfeeding from these 10 items (scale 0–10; mean of 4.7; median of 4), in which one point was added to the score for each benefit mentioned. We plotted the prevalence of EBF by each awareness score to determine a cut‐off point for good awareness of the benefits of breastfeeding. The result showed a score of six indicated good awareness. Twenty‐two per cent of the women had an awareness score of 6–10.

We also asked mothers about the environment related to breastfeeding practices. The environmental factors included (i) whether her partner agrees that breastmilk is better than BMS, (ii) having received information that encourages breastfeeding, (iii) exposure to infant formula advertisements and free samples and (iv) feelings of inconvenience when breastfeeding in public and the use of infant formula due to this inconvenience. For formally employed women, we asked about the availability of refrigerators at work to store breastmilk and if they have returned to work.

#### 2.2.3 Covariate variables

We collected data on maternal characteristics such as age (categorized into 18–25, 26–35 and ≥36 years), ethnicity (Han and other ethnicities), education (junior high school or less [<9 years], high school [9–12 years] and college or higher education), employment status (formally employed and not formally employed) and the place of residence (big cities, medium and small cities and rural areas). A mother was classified as formally employed if she responded that she was formally employed and covered by the paid maternity leave policy. We asked mothers if they had had an antenatal visit and if they had experienced gestation diabetes or high blood pressure during their pregnancy. We also collected information about child characteristics and delivery‐related factors including gender, age in months, first birth, low birthweight or caesarean delivery. We also asked the mothers if the child had had diarrhoea or a symptom of respiratory infection within 2 weeks prior to the interview.

### 2.3 Statistical analysis

From 10,408 records of interviewed mothers, we excluded 185 migrant women in rural areas because the study protocol only included locally registered residents in rural areas. We also excluded 12 records with missing values for outcomes (e.g., ever breastfed, EBF and EIBF), 397 records for exposure variables (e.g., knowledge, social norms and the environment relating to breastfeeding) or covariates relating to childbirth (e.g., first birth, caesarean delivery and low birthweight), and 69 records due to missing information on maternal characteristics (e.g., age, ethnicity and employment status). The sample size for the analysis was 9,745, which is about 95% of the original sample. Sensitivity analysis using the complete sample showed very similar findings with those reported in this manuscript.

We used survey commands in Stata 14.1 (Stata Inc., TX, USA) to consider sampling weight and survey design effects (i.e., cluster effect within strata [district/county] and cluster [township/street]). The sampling weight was estimated and provided by NINH, China CDC as an inverse number of the overall probability to be selected for the study with an attempt to provide representative data for all children from 0 to 11 months old in China. First, we performed weighted descriptive analysis on the background information of the mothers and infants as well as breastfeeding practices and associated factors. We then examined breastfeeding patterns and stratified them by women's employment status. Second, we performed multiple logistic regressions to examine the associated factors of each breastfeeding practice. For ever breastfed and EIBF, the independent variables were place of residence, child gender, factors relating to delivery and maternal characteristics. For EBF and breastfeeding on the previous day, the independent variables were the place of residence, child gender, factors relating to delivery, maternal characteristics, previous breastfeeding practices, maternal knowledge about breastfeeding and the environment relating to breastfeeding. We further stratified the logistic regression analysis for EBF and breastfeeding on the previous day by employment status of the mothers because formally employed women might have different characteristics such as maternity leave, workplace support, income and socio‐economic status compared with unemployed or informally employed women. For formally employed mothers, we also examined the benefits of not yet having returned to work and access to a refrigerator to store breastmilk at the workplace on exclusive and continued breastfeeding.

### 2.4 Ethical considerations

The study design was reviewed and approved by the Medical Research Ethnics Committee at the National Institute for Nutrition and Health at the Chinese Center for Disease Control and Prevention (NINH, China CDC). Written informed consents were obtained from all participants.

## 3 RESULTS

Among 9,745 mothers interviewed, 89.6% belonged to the Han ethnicity, 28.1% had at least a college degree and 16.6% had formal employment (Table 1). About 50.5% of the infants were boys, 38.7% were the first child and 42.0% were born by caesarean delivery. Mothers had a moderate level of breastfeeding knowledge (Table 1). Three‐quarters of women received information that encouraged breastfeeding, and 89.6% had a partner who agreed that breastfeeding was better than BMS. However, breastfeeding in public places was not a norm, 28.2% of the women were advised by health staff, mass media or social networks about using infant formula, and 18.3% received free infant formula samples (Table 1). Less than half of the formally employed mothers had refrigerators at work to store breastmilk (Table 1).

**TABLE 1:** Demographics of mother–child dyads and studied knowledge and practices^a^

	% or mean (*n* = 9,745)
Place of residence	
Big cities	8.5
Medium and small cities	46.9
Rural areas	44.5
Factors relating to the child and delivery	
The child was male	50.5
The first child	38.7
Mean child age (months)	5.4
Mother had antenatal visits	96.8
Caesarean delivery	42.0
Child had ever had disease 2 weeks prior to the interview	31.2
Low birthweight	4.7
Gestation diabetes or high blood pressure during pregnancy	7.8
Other breastfeeding practices	
Correct latching	90.1
Early initiation of breastfeeding	8.2
Maternal characteristics	
Belong to Han ethnicity	89.6
Age	
≤25 years	24.6
26–35 years	65.1
36+ years	10.2
Mother education group	
Junior high school or less	50.0
High school	22.0
College or higher education	28.1
Formally employed	16.6
Maternal knowledge about breastfeeding	
Knew the time mother could produce colostrum	76.0
Knew that a baby should be exclusive breastfed in the first 6 months	56.4
Knew the best way to stimulate milk production was to nurse more	84.5
Knew that a baby should be breastfeed on demand	79.6
Good awareness on the benefits of breastfeeding (≥ 6 correct responses out of 10)^b^	22.6
Environment relating to breastfeeding	
Partner agreed that breastfeeding was better than breastmilk substitutes	89.6
Received information that encourages breastfeeding	75.2
Received advice to feed the baby with infant formula	28.2
Received free infant formula samples	18.3
Fed the baby with infant formula due to inconvenience of breastfeeding in public places	22.1
Felt inconvenient to breastfeed in public places	75.0
Had refrigerator at the workplace to store breastmilk (among formally employed women; *n* = 1,334)	39.0
Had already returned to work (among formally employed women; *n* = 1,334)	15.9

a Data were weighted prevalence or mean; (*n*) was for nonweighted sample size.

b Score for awareness on the benefits of breastfeeding: mean: 4.7; median: 4; range: 0–10.

**FIGURE 1:**
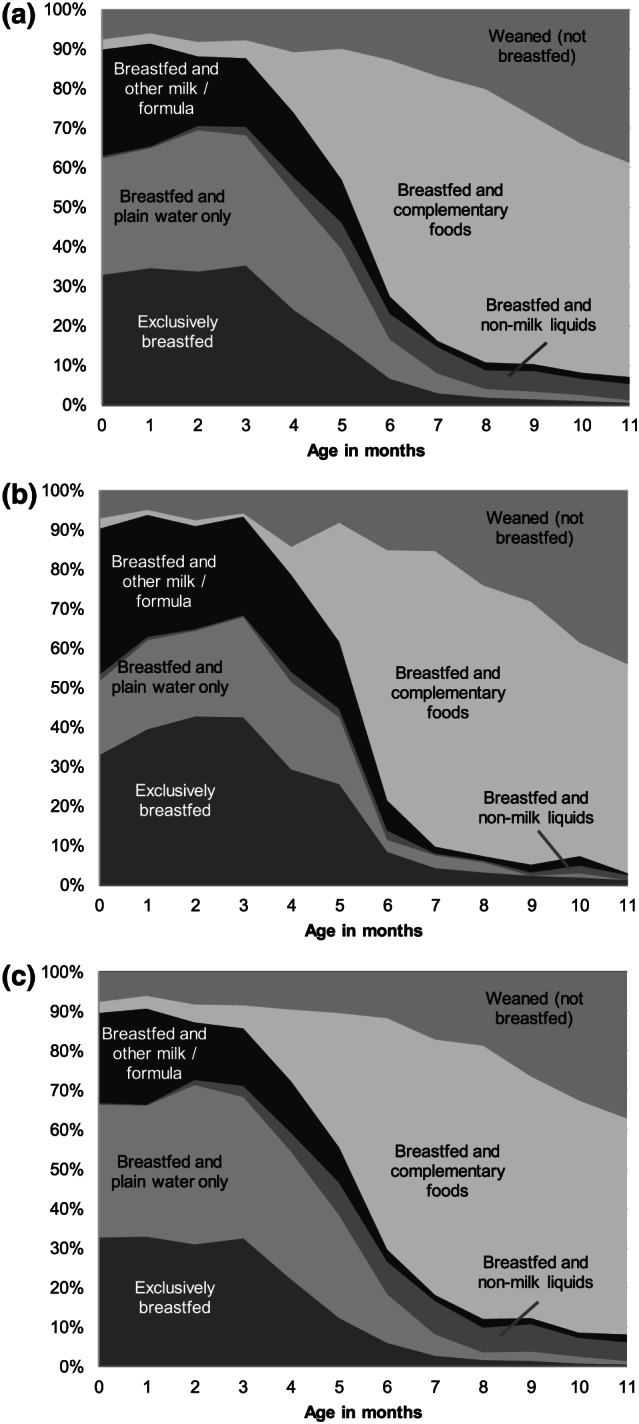
Breastfeeding pattern for infants from (a) all mothers (*n* = 9,745), (b) formally employed mothers (*n* = 2485) and (c) not formally employed mothers (*n* = 7260). Data were weighted prevalence; (*n*) was for nonweighted sample size

Almost all (97.4%; 95% CI: 96.4, 98.5) children 0–11 months old in this sample had been breastfed at some point (i.e., ever breastfed); however, only 8.2% (95% CI: 6.3, 10.2) were breastfed within 1 h after birth. The prevalence of EBF in children 0–5 months old was 27.8% (95% CI: 25.4, 30.3), which dropped from 31.0% (95% CI: 28.5, 33.5) for 0–3 months to 25.9% (95% CI: 20.8, 31.0) for 4 months and to 16.7% (95% CI: 12.3, 21.1) for 5 months (Figure 1). Feeding plain water and infant formula were the main barriers to EBF. The prevalence of EBF was 29.8% (95% CI: 24.2, 35.3) in formally employed mothers and 27.4% (95% CI: 24.9, 30.0) among mothers who were not formally employed. Feeding with infant formula was the key barrier to EBF among formally employed women, whereas giving water was the key barrier among other mothers (Figure 1).

The prevalence of ever‐breastfeeding was lower among first‐child mothers compared with those with a subsequent child (OR: 0.37; 95% CI: 0.25, 0.53), mothers with a low birthweight child compared with mothers with a normal birthweight child (OR: 0.41; 95% CI: 0.19, 0.89) and mothers with gestational diabetes or high blood pressure during pregnancy (OR: 0.59; 95% CI: 0.37, 0.95; Table 2). EIBF was less likely in cases of caesarean delivery (OR: 0.52; 95% CI: 0.42, 0.65; Table 2). EIBF was more likely in mothers with higher than lower education and those of Han ethnicity compared with other ethnicities (Table 2).

**TABLE 2:** Associated factors of ever breastfed and early initiation of breastfeeding^a^

	Ever breastfed	Early initiation of breastfeeding
	(*n* = 9,745)	(*n* = 9,745)
Factors relating to the delivery		
The first child	0.37^***^ (0.25, 0.53)	0.77 (0.57, 1.04)
Mother had antenatal visits	1.19 (0.28, 5.12)	1.40 (0.93, 2.09)
Caesarean delivery	0.63 (0.33, 1.20)	0.52^***^ (0.42, 0.65)
Low birthweight	0.41^*^ (0.19, 0.89)	0.46 (0.14, 1.51)
Gestation diabetes or high blood pressure during pregnancy	0.59^*^ (0.37, 0.95)	1.15 (0.69, 1.93)
Maternal characteristics		
Belong to Han ethnicity	0.84 (0.30, 2.37)	3.26^***^ (1.81, 5.87)
Age (reference: ≤25 years)		
26–35 years	0.48 (0.23, 1.02)	0.99 (0.76, 1.29)
36+ years	0.31 (0.09, 1.02)	0.78 (0.60, 1.00)
Education group (reference: Junior high school or less)		
High school	1.69 (0.92, 3.11)	0.95 (0.71, 1.27)
College or higher education	1.17 (0.67, 2.04)	1.38^*^ (1.02, 1.88)
Formally employed	1.42^*^ (1.01, 2.00)	1.11 (0.66, 1.86)

a Values are weighted, adjusted odds ratios (OR) and 95% CI from survey multiple logistic regression, controlled for place of residence and child gender. Significantly different from the null value (OR of 1). (*n*) was for nonweighted sample size.

* *P* < 0.05,

** *P* < 0.01,

*** *P* < 0.001.

The prevalence of EBF under 6 months was lower in medium and small cities as well as rural areas when compared with big cities (Table 3). It was lower in mothers with caesarean delivery (OR: 0.79; 95% CI: 0.66, 0.94) and children with diarrhoea or a symptom of respiratory infection 2 weeks prior to interview (OR: 0.68; 95% CI: 0.56, 0.84; Table 3). The prevalence of EBF was higher among mothers who practiced EIBF, had received information that encouraged breastfeeding and who knew that a baby should be breastfed on demand and exclusively (Table 3). In contrast, the prevalence of EBF was lower in mothers receiving infant formula advice or feeling unease breastfeeding in public places (Table 3). There were some differences in the factors associated with EBF between mothers who were formally employed and those who were not, such as the child age, caesarean delivery, breastfeeding practices, EIBF and receiving advice about infant formula (Table 3).

**TABLE 3:** Associated factors of exclusive breastfeeding under 6 months^a^

	All women	Formally employed women	Not formally employed women
	(*n* = 4,966)	(*n* = 1,334)	(*n* = 3,632)
Place of residence			
Medium and small cities	0.48^**^ (0.31–0.75)	0.41^***^ (0.25–0.68)	0.64 (0.35–1.18)
Rural areas	0.63^*^ (0.41–0.97)	0.48 (0.16–1.40)	0.82 (0.43–1.55)
Factors relating the child and delivery			
The first child	0.93 (0.78, 1.10)	0.90 (0.63, 1.28)	0.94 (0.77, 1.14)
Age in months	0.93^*^ (0.87, 1.00)	1.07 (0.96, 1.19)	0.91^**^ (0.85, 0.97)
Mother had antenatal visits	1.24 (0.77, 1.98)	1.70 (0.81, 3.59)	1.10 (0.67, 1.81)
Caesarean delivery	0.79^**^ (0.66, 0.94)	0.75 (0.48, 1.17)	0.79^**^ (0.66, 0.93)
Child had ever had disease 2 weeks prior to the interview	0.68^***^ (0.56, 0.84)	0.72 (0.39, 1.33)	0.69^***^ (0.56, 0.83)
Previous breastfeeding practices			
Correct latching	1.43 (0.98, 2.08)	3.76^*^ (1.34, 10.55)	1.19 (0.83, 1.71)
Early initiation of breastfeeding	1.35^*^ (1.04, 1.77)	2.02^**^ (1.34, 3.04)	1.21 (0.90, 1.63)
Maternal knowledge about breastfeeding			
Knew the time mother could produce colostrum	1.11 (0.88, 1.39)	1.03 (0.58, 1.84)	1.13 (0.85, 1.50)
Knew that a baby should be exclusive breastfed in the first 6 months	1.23^*^ (1.05, 1.45)	1.42 (0.84, 2.40)	1.22^*^ (1.02, 1.46)
Knew the best way to stimulate milk production was to nurse more	0.95 (0.72, 1.25)	0.75 (0.30, 1.87)	0.98 (0.75, 1.28)
Knew that a baby should be breastfeed on demand	1.53^**^ (1.18, 1.99)	1.05 (0.65, 1.68)	1.65^**^ (1.22, 2.24)
Good awareness on the benefits of breastfeeding	1.17 (0.84, 1.64)	1.77^**^ (1.20, 2.61)	1.02 (0.66, 1.59)
Environment relating to breastfeeding			
Partner agreed that breastfeeding was better than breastmilk substitutes	1.11 (0.75, 1.65)	1.18 (0.67, 2.05)	1.14 (0.70, 1.86)
Received information that encourages breastfeeding	1.33^*^ (1.02, 1.75)	0.85 (0.37, 1.94)	1.42^*^ (1.06, 1.91)
Received advice to feed the baby with infant formula	0.52^***^ (0.41, 0.65)	0.63 (0.39, 1.03)	0.48^***^ (0.35, 0.67)
Received free infant formula samples	1.26 (0.91, 1.73)	1.11 (0.76, 1.63)	1.32 (0.93, 1.86)
Fed the baby with infant formula due to inconvenience of breastfeeding in public	0.62^**^ (0.46, 0.83)	0.52^***^ (0.36, 0.76)	0.64^*^ (0.42, 0.97)
Felt inconvenient to breastfeed in public	0.78 (0.60, 1.03)	1.25 (0.82, 1.90)	0.74 (0.55, 1.00)
Formally employed	1.11 (0.81, 1.54)		
Had refrigerator at the workplace to store breastmilk		1.33 (0.95, 1.86)	
Had already returned to work		0.69 (0.43, 1.11)	

a Values are weighted, adjusted odds ratios (OR), and 95% CI from survey multiple logistic regression models, controlled for maternal age, education and ethnicity, and child gender. Significantly different from the null value (OR of 1). (*n*) was for nonweighted sample size.

* *P* < 0.05.

** *P* < 0.01.

*** *P* < 0.001.

The prevalence of receiving breastmilk on the previous day among infants aged 6–11 months was 77.5%. This prevalence was lower for the first child (OR: 0.72; 95% CI: 0.54, 0.98) and if the child had diarrhoea or a symptom of respiratory infection 2 weeks prior to the interview (OR: 0.81; 95% CI: 0.66, 1.00; Table 4). The prevalence of breastfeeding on the previous day was higher in mothers whose partners supported breastfeeding and who knew about the timing of colostrum production, EBF for 6 months, and to nurse more to stimulate milk production. The prevalence was lower in mothers receiving infant formula advice or feeling uneasy breastfeeding in public places (Table 4).

**TABLE 4:** Associated factors of breastfeeding on the previous day for 6–11 months old^a^

	All women	Formally employed women	Not formally employed women
	(*n* = 4,804)	(*n* = 1,160)	(*n* = 3,644)
Factors relating the child and delivery			
The first child	0.72^*^ (0.54, 0.98)	1.80 (0.85, 3.82)	0.60^**^ (0.44, 0.82)
Age in months	0.77^***^ (0.70, 0.85)	0.72^***^ (0.63, 0.82)	0.78^***^ (0.70, 0.87)
Mother had antenatal visits	0.59^*^ (0.40, 0.89)	0.21^**^ (0.07, 0.66)	0.66 (0.42, 1.03)
Caesarean delivery	1.06 (0.80, 1.41)	1.10 (0.80, 1.50)	1.10 (0.80, 1.51)
Child had ever had disease 2 weeks prior to the interview	0.81^*^ (0.66, 1.00)	0.80 (0.52, 1.23)	0.79^*^ (0.64, 0.97)
Previous breastfeeding practices			
Correct latching	1.76^**^ (1.22, 2.53)	2.03 (0.97, 4.25)	1.70^*^ (1.14, 2.55)
Early initiation of breastfeeding	1.06 (0.65, 1.73)	0.69 (0.36, 1.35)	1.31 (0.77, 2.24)
Maternal knowledge about breastfeeding			
Knew the time mother could produce colostrum	1.72^**^ (1.19, 2.48)	0.94 (0.56, 1.59)	1.92^**^ (1.30, 2.84)
Knew that a baby should be exclusive breastfed in the first 6 months	1.34^**^ (1.08, 1.67)	1.16 (0.65, 2.07)	1.36^**^ (1.08, 1.70)
Knew the best way to stimulate milk production was to nurse more	1.40^*^ (1.06, 1.83)	2.20^*^ (1.17, 4.16)	1.29 (0.88, 1.89)
Knew that a baby should be breastfeed on demand	1.18 (0.91, 1.53)	0.98 (0.59, 1.62)	1.22 (0.94, 1.58)
Good awareness on the benefits of breastfeeding	0.73^*^ (0.55, 0.97)	1.37 (0.81, 2.34)	0.61^**^ (0.46, 0.83)
Environment relating to breastfeeding			
Partner agreed that breastfeeding was better than breastmilk substitutes	1.65^***^ (1.24, 2.19)	1.31 (0.61, 2.81)	1.78^***^ (1.33, 2.40)
Received information that encourages breastfeeding	1.22 (0.92, 1.62)	1.78 (0.92, 3.45)	1.18 (0.85, 1.65)
Received advice to feed the baby with infant formula	0.59^***^ (0.47, 0.74)	0.95 (0.59, 1.51)	0.52^***^ (0.40, 0.68)
Received free infant formula samples	1.07 (0.74, 1.55)	1.79 (0.90, 3.58)	0.98 (0.69, 1.40)
Fed the baby with infant formula due to inconvenience of breastfeeding in public	0.52^***^ (0.38, 0.69)	0.57^*^ (0.36, 0.90)	0.50^***^ (0.35, 0.71)
Felt inconvenient to breastfeed in public	1.05 (0.79, 1.39)	0.63 (0.31, 1.29)	1.15 (0.83, 1.58)
Formally employed	0.83 (0.57, 1.19)		
Had refrigerator at the workplace to store breastmilk		1.08 (0.63, 1.86)	
Had already returned to work		0.86 (0.51, 1.45)	

a Values are weighted, adjusted odds ratios (OR) and 95% CI from survey multiple logistic regression models, controlled for place of residence, maternal age, education and ethnicity, and child gender. Significantly different from the null value (OR of 1). (*n*) was for nonweighted sample size.

* *P* < 0.05.

** *P* < 0.01.

*** *P* < 0.001.

## 4 DISCUSSION

In this study, we found that breastfeeding practices were inadequate in 12 districts/counties in China. The prevalence of EIBF found in our study (8.2%) is lower than the national prevalence reported in 2013 of 28.7% (UNICEF, 2018). The prevalence we reported is similar to a study in Sichuan province of 9% (Tang et al., 2013) but much lower than a study in central and western China of 59.4% (Guo et al., 2013). Given that the prevalence of EIBF varied substantially by region in China, the difference in sampling strategies among studies might partially contribute to the differences observed. The prevalence of EBF in China from previous surveys was 27.6% in 2008 (Center for Health Statistics and Information at Ministry of Health of People's Republic of China, 2009), 20.7% (crude) and 18.6% (weighted) in 2013 (Duan et al., 2018) and 27.8% from our study. The prevalence was lower than the world average (42%) and about the same as in the East Asia and the Pacific Region (28%; UNICEF, 2019). The prevalence of EBF was lower than the national target for EBF of 50% (General Office of the State Council of the People's Republic of China, 2017).

Various factors from individual to national levels seem to be associated with inadequate breastfeeding practices in China. First, at the individual level, our study showed that breastfeeding knowledge was associated with a higher prevalence of EBF among infants 0–5 months old and continued breastfeeding in children 6–11 months old. Previous studies showed that breastfeeding education helped to improve breastfeeding knowledge and breastfeeding practices (Oche, Umar, & Ahmed, 2011; Radhakrishnan & Balamuruga, 2014; Shi et al., 2008). Breastfeeding beliefs, knowledge and self‐efficacy can also be improved through interpersonal communications and mass media campaigns (Nguyen et al., 2016; Nguyen et al., 2017).

Second, at the household level, we found that mothers with support from partners (e.g., proxy through the support of breastfeeding over BMS) were more likely to continue to breastfeed their infants. In China, especially in urban areas, there has been a shift from multiple generation households to two‐generation households (Su, Hu, & Peng, 2017). Among migrants, the simultaneous migration of the couple has become the dominant form of migration for young couples (Meng, Zhao, & Liwu, 2016). Although taking care of newborns is considered to be the responsibility of the extended family in China, conflicts often happen among new mothers, their mothers and mothers‐in‐law about how to take care of babies, especially regarding breastfeeding (Tang, Zhu, & Zhang, 2016). To avoid such conflicts, young families might choose to separate from their extended families to have full autonomy over their decisions about child feeding and other concerns (Tang et al., 2016). The shift might reduce the influence of mothers or mothers‐in‐law in the decision making and increase the influence of the partner for child feeding practices. A recent review of randomized controlled trials and quasi‐experimental studies showed that the inclusion of fathers or partners in breastfeeding interventions improves EIBF, EBF and continued breastfeeding prevalence (Abbass‐Dick, Brown, Jackson, Rempel, & Dennis, 2019).

Third, for factors at the health facility level, we found that the prevalence of caesarean delivery was high and associated with a lower prevalence of EIBF. The prevalence of caesarean deliveries in our study, 42%, was comparable with previous studies (Feng, Xu, Guo, & Ronsmans, 2012; UNICEF, 2019) and much higher than the WHO's suggested prevalence between 10% and 15% (Betran, Torloni, Zhang, & Gülmezoglu, 2016). This finding suggests the need of regulating the indication of caesarean delivery due to nonclinical reasons, which is high and affected by factors from women and family members, health workers, the health system and socio‐cultural norms (Feng et al., 2012; Long et al., 2018). Although the WHO issued guidelines for early essential newborn care for both vaginal and caesarean deliveries (Betran et al., 2016), current childbirth and early essential newborn care policy and practice in China do not align with the recommendation from the WHO according to a recent study in four provinces in China (Xu, Yue, Wang, Murray, & Sobel, 2018). It is essential for maternal and child health facilities in China to adopt more breastfeeding‐friendly policy and practices at health facilities. This adoption at scale would have a large impact on improved EIBF and EBF at discharge in China—a country with a very high coverage of antenatal, delivery and postnatal care at health facilities (UNICEF, 2019).

Fourth, related to the Code compliance, we found that receiving advice on using BMS was associated with lower EBF and continued breastfeeding prevalence. Code violations have been reported earlier in China, including BMS advertisement, labelling and provision of free BMS samples (Liu, Dai, Xie, & Chen, 2014). Studies in China, India and Vietnam have shown that the effectiveness of the Code is dependent on countries' engagement with implementation strategies and the presence of other enabling factors (Robinson, Buccini, Curry, & Perez‐Escamilla, 2019). However, the situation is challenging in China, a country with almost 17 million births annually (UNICEF, 2019) and the world's largest market for BMS (Rollins et al., 2016). China promulgated *Administrative Measures on Marketing of Breast‐milk Substitutes* in 1995 that followed the Code. Between 1995 and 2017, the supervision and enforcement of the legislation were insufficient and not regularly updated to capture new marketing tactics of BMS companies. As of the end of 2017, this regulation was abolished without any replacement, leaving the BMS market and marketing unregulated (National Health and Family Planning Commission of the People's Republic of China, 2017). In addition, the introduction of Decree 26 *Administrative Measures for the Registration of Recipes for Formula Powder Products for Infants and Young Children* (China Food and Drug Administration, 2016) leaves the market wide open for reputable brands that used to be available mostly in China's big cities to invade medium and small cities and rural areas, replacing substandard products in the market (Chen & Hu, 2017). As a result, the sale of baby food—about 90% for formula milk—increased by 104% during 2010–2014 and by an additional 47% during 2014–2018 to reach CNY 182.3 billion (or USD 26.5 billion; conversion rate: 1 CNY = 0.14561 US$ on September 15, 2018; Euromonitor International, 2015, 2018).

Fifth, our study found that lactating women might feel unease breastfeeding in public places, which suggests the need for an environment that is more breastfeeding friendly. With the successful implementation of the ‘universal two‐child’ policy in China, there would be a significant increase in the demand for lactation rooms in public places such as airports, railway stations, shopping malls and tourist centres. Our findings showed that 90% of the mothers in the sample thought that there were not enough lactation rooms in their living areas, and 22.1% of them opted to feed their children with infant formula because of the inconvenience of breastfeeding in public places.

Sixth, our study also suggests the need for better maternity protection and workplace breastfeeding support in China, a country with 70% of women participating in the labour force (Ye & Zhao, 2018). In our sample, only 16.6% of the women interviewed were formally employed and eligible for paid maternity leave. We found that the child's age did not predict EBF in formally employed mothers, while it was associated with a lower prevalence of EBF among those mothers who were not formally employed. Because paid maternity leave protects women's employment and income during their pregnancy and after childbirth, the women are more likely to have better breastfeeding practices (Chai, Nandi, & Heymann, 2018; Rollins et al., 2016). Previous studies showed that mothers who returned to work were less likely to practice EBF (Jia, Dong, & Song, 2018; Mirkovic, Perrine, & Scanlon, 2016). Given that the duration of paid maternity leave in China is just 14 weeks, it is challenging for mothers to exclusively breastfeed their children for 6 months. Additionally, mothers need lactation support at work, including nursing breaks and refrigerators for storing breastmilk (International Labour Organization, 2014). In our study, we found that simple interventions, such as the availability of a refrigerator to store breastmilk in the workplace, were potentially associated with higher EBF (OR: 1.33; 95% CI: 0.95, 1.86). These findings suggest that legislation and interventions are needed to make the workplace a more supportive environment for breastfeeding mothers.

Our study examined factors associated with breastfeeding practices at multiple levels in China using a large sample drawn from all seven regions and included 12 of 34 provincial level administrative divisions, covering large cities, medium and small cities and rural areas. The sample size was large enough to stratify the analyses by the working status of the women. The study has limitations as well. Because the selection of provinces and districts/counties was not random, our results might only apply to study sites. The recruitment of mothers from vaccination clinics would provide comparable findings with those from households because of a strong immunization registry and the immunization programme's high coverage in China (99%; UNICEF, 2019). Because the respondent was the mother, we excluded children who were not with their mothers at the time of the survey, including ‘children left behind’ (Tian, Ding, Shen, & Wang, 2017), which might overestimate the prevalence of EBF and breastfeeding on the previous day. Due to the limitations in sampling, we might expect a random variation in the prevalence of ever breastfed and EIBF from the ‘true’ value. The cross‐sectional design cannot be used to conclude causal relationships. However, plausibility of causality was increased in our study by selecting age ranges for which exposure or covariate variables occurred before or at about the same time as the practices. We did not collect information about the intermediate stages between awareness and practices (e.g., intentions, trials, adoption and maintenance) because they were not the focus of this study. Also, although we capture key information relating to BMS and maternity protection, the content of our questionnaire was not as exhaustive as the complete list of the WHO's NetCode Assessment Module and International Labor Organization's Maternity Protection Assessment Toolkit. Nonetheless, we captured key individual, health facility and policy components.

In conclusion, we found that the prevalence of EIBF and EBF practices in China were low and associated with factors at the individual, family, health facility and environmental levels. Interventions to improve breastfeeding practices in China should include improved breastfeeding knowledge and skills by mothers and other caregivers, and breastfeeding‐friendly environments in health facilities, and public places. In addition, improved maternity protection legislation and national regulations aligned with the Code seem critical to protect breastfeeding and ensure the health and well‐being of children and women in China. To achieve the above, China should build on the evidence of successful experiences, tools and platforms for breastfeeding protection, promotion and support in the region and in the world (Rollins et al., 2016; UNICEF, 2019).

## CONFLICTS OF INTEREST

The authors declare that they have no conflicts of interest.

## CONTRIBUTIONS

The authors' responsibilities were as follows: JL, TTN, XW and JF designed the study; JL, TTN and XW acquired and analysed the data; JL, TTN, XW and RM interpreted the data; JL, TTN and XW drafted this manuscript; and JL, TTN, XW, RM and JF provided critical intellectual feedback to help revise the manuscript. All authors have read and approved the final manuscript.
